# Unveiling the Potential: A Comprehensive Review of Dynamic Slow-Motion Video Endoscopy for Eustachian Tube Dysfunction Evaluation

**DOI:** 10.7759/cureus.63811

**Published:** 2024-07-04

**Authors:** Nimisha Patil, Shraddha Jain, Smriti Wadhwa

**Affiliations:** 1 Otolaryngology - Head and Neck Surgery, Jawaharlal Nehru Medical College, Datta Meghe Institute of Higher Education and Research, Wardha, IND

**Keywords:** clinical applications, otologic conditions, management, diagnosis, dynamic slow motion video endoscopy, eustachian tube dysfunction

## Abstract

Eustachian tube dysfunction (ETD) poses diagnostic challenges due to its complex pathophysiology and varied clinical presentation. Traditional diagnostic methods often lack direct visualization of the Eustachian tube (ET) function, leading to suboptimal evaluation and management. Dynamic slow-motion video endoscopy (DSVE) has emerged as a novel approach to address these limitations, offering real-time visualization of ET dynamics with enhanced clarity and precision. This comprehensive review provides an overview of DSVE as a promising tool for evaluating ETD. We discuss its methodology, clinical applications, comparative analysis with traditional methods, and future directions. Key findings from the literature highlight DSVE's ability to enhance diagnostic accuracy, facilitate targeted treatment strategies, and improve patient outcomes. Integrating DSVE into routine clinical practice holds significant implications for the diagnosis and management of ETD, offering clinicians valuable insights into underlying pathophysiology and guiding personalized treatment interventions. Future research should focus on standardizing DSVE protocols, validating its diagnostic accuracy, and exploring its role in guiding novel treatment modalities. By advancing our understanding of ETD and optimizing diagnostic and therapeutic approaches, DSVE has the potential to revolutionize the management of this common yet challenging otologic condition.

## Introduction and background

The Eustachian tube (ET) plays a crucial role in middle ear health, and its dysfunction (Eustachian tube dysfunction (ETD)) remains a significant challenge in otology due to the region's limited accessibility. ETD is recognized as the primary contributing factor in the development of middle ear diseases. However, there is currently no single test that comprehensively evaluates both the anatomical and physiological functions of the ET. Most endoscopic investigations have primarily focused on static examinations of the ET orifice or lumen, rather than dynamic monitoring of tubal motion. Dynamic slow-motion video endoscopy (DSVE) is a relatively novel approach for assessing ET dilatory movements. This comprehensive review provides an overview of DSVE as a promising tool for evaluating ETD. It discusses the methodology, clinical applications, comparative analysis with traditional methods, and future directions of this technique. Key findings from the literature highlight DSVE's ability to enhance diagnostic accuracy, facilitate targeted treatment strategies, and improve patient outcomes in ETD management. Integrating DSVE into routine clinical practice holds significant implications for the diagnosis and management of ETD, offering clinicians valuable insights into underlying pathophysiological mechanisms and guiding personalized treatment interventions. Future research should focus on standardizing DSVE protocols, validating its diagnostic accuracy, and exploring its role in guiding novel treatment modalities. By advancing our understanding of ETD and optimizing diagnostic and therapeutic approaches, DSVE has the potential to revolutionize the management of this common yet challenging otologic condition.

## Review

The ET plays a pivotal role in regulating pressure equilibrium between the middle ear and the nasopharynx, ensuring optimal ventilation of the middle ear cavity. Inflammation of the ET impedes its ability to facilitate gas exchange between the middle ear and the nasopharynx, resulting in auditory impairment or a sensation of aural fullness. Irrespective of causality, tubal inflammation precipitates an inability to effectively clear middle ear secretions, leading to stasis and predisposing to the development of otitis media. ETD poses diagnostic challenges due to its intricate pathophysiological mechanisms and diverse clinical manifestations [1]. Conventionally, the assessment of ETD has relied on subjective patient reports, otoscopic examination, tympanometry, and audiometry, which often fail to provide direct visualization of ET function and may not accurately capture dynamic changes within the tubal lumen [2]. Recognizing the limitations of traditional diagnostic modalities, DSVE has emerged as a promising technique for evaluating ETD. DSVE facilitates real-time visualization of ET dynamics with enhanced clarity and precision, offering insights into the underlying mechanisms of dysfunction and enabling differentiation between mechanical and functional etiologies, thereby facilitating targeted therapeutic interventions [3]. This review aims to provide a comprehensive overview of DSVE as a novel approach for evaluating ETD. By synthesizing existing literature and discussing clinical applications, methodology, comparative analysis with traditional methods, and future directions, this review seeks to elucidate the potential of DSVE in advancing the diagnosis and management of ETD.

Traditional techniques to assess ETD

The progression of endoscopic evaluation techniques has witnessed substantial advancements over time. Traditional approaches have evolved into more sophisticated and minimally invasive procedures, offering enhanced diagnostic and therapeutic capabilities. Several techniques have been developed recently to evaluate tubal function. Advances in radiologic imaging and the widespread use of fibreoptic endoscopes have provided a more detailed understanding of the ET's structure and function [4]. However, otologists still find it difficult to distinguish between anatomical and physiological dysfunctions of the ET, despite the availability of various tubal function tests [4]. A single technique that assesses anatomical and physiological aspects has yet to be developed [5].

Otoscopy

ETD was initially assessed clinically by otoscopy, which revealed features like a retraction of pars tensa and pars flaccida and mobility of the tympanic membrane (TM) by performing Valsalva and Toynbee maneuvers to assess the patency. Still, their reliability is limited, as up to one-third of healthy individuals may fail these tests [5]. While these otoscopic findings provide some insight into middle ear pressure and ET patency, they are not sensitive measures of the ET's current physiological performance [5].

Manometric Tests

Manometric tests are tools designed to evaluate the ventilatory and pressure equalization abilities of the ET with intact TM. Tympanometry, part of immittance audiometry, measures middle ear pressure and detects middle ear effusions with high sensitivity and specificity. However, it has limitations, such as failing to detect subtle anatomical abnormalities and fluctuating middle ear pressures [5]. Simple manometric tests like the Valsalva and Toynbee maneuvers can indicate ET patency, but their reliability is variable. Advanced tests such as tubo-tympano-aerodynamic-graphy (TTAG) and formal manometric tests, including the inflation-deflation test, forced response test, and nine-step test, offer more controlled and detailed assessments that measure both active and passive ET functions, with varying degrees of sensitivity and specificity. The inflation-deflation test evaluates active ET function by applying positive and negative pressures to the middle ear. In contrast, the forced response test measures passive ET opening by applying steady pressure until the ET opens. The nine-step test assesses ET function with an intact TM by inducing middle ear pressure changes through external canal pressure variations. Combining multiple manometric test parameters can improve diagnostic accuracy for ET dysfunction. Tympanometry can also detect patulous ET by identifying respiratory-synchronous compliance patterns in the ear canal pressure trace.

Sonotubometry

Sonotubometry (STM) evaluates the physiological assessment of ET function in a non-invasive method, using a sound source applied to the nostril and a microphone in the external auditory canal, and detects increased sound levels when the ET opens during swallowing. However, it is not always effective in detecting middle ear effusions, and about 10-20% of healthy individuals do not show detectable ET opening during swallowing. Combining STM with other tests, like the nine-step test, improves its diagnostic utility [5].

Tubomanometry

Tubomanometry (TMM) involves the patient sitting with a pressure receiver in the external auditory canal and a nasal applicator to create a defined nasopharyngeal pressure. The patient swallows, triggering a synchronized pressure increase in the nasopharynx. TMM assesses both active and passive ET openings and records pressure changes in the nasopharynx and middle ear, but no direct visualization of the ET [5]. 

Impedance Audiometry 

Impedance audiometry is the most used method to evaluate middle ear pressure regulation in patients with middle ear diseases [5]. Although tympanometry is an objective test, there is considerable variability in the results from different tympanometry-based ET function tests, and it often fails to detect subtle anatomical abnormalities that lead to functional deficits [5].

Limitations of Traditional Endoscopy in ETD Diagnosis

Traditional endoscopic evaluation techniques often fall short when it comes to diagnosing ETD. Most evaluations of ET dysfunction fail to distinguish the type of dysfunction between mechanical and functional types of dysfunction [6]. Due to the relative inaccessibility of the ET and the significant variability in results among different tympanometric tests for ET function, the search for an optimal method of assessing ET dysfunction continues [6]. However, DSVE has emerged as a promising solution to address these limitations. Numerous studies have underscored DSVE's potential to identify and classify pathologic changes within the ET, providing crucial insights into structural and functional abnormalities [7].

Introduction of High-Speed and Slow-Motion Endoscopy

Introducing high-speed and slow-motion endoscopy is a significant leap forward in evaluation techniques. These cutting-edge technologies offer unprecedented insights into the dynamic behavior of various anatomical structures, revolutionizing the diagnostic and treatment planning processes. High-speed video endoscopy (HSV) stands out as a remarkable innovation, allowing for the capture of vocal fold vibrations at an exceptionally high frame rate, often surpassing 2000 frames per second [8,9]. By detecting subtle abnormalities in vocal fold vibration patterns, HSV contributes to diagnosing both benign and malignant lesions, thereby improving clinical outcomes [9]. Similarly, dynamic slow-motion video endoscopy (DSVE) represents another milestone in endoscopic evaluation techniques. DSVE captures endoscopic images at a high frame rate, followed by playback at a slower speed, facilitating meticulous observation of dynamic processes [10]. This versatile technique finds application across various fields evaluating ETD in chronic otitis media patients [9].

*Advantages of Dynamic Slow-Motion Video* *Over Traditional Techniques*

Enhanced visualization is a hallmark feature of DSVE, as it captures endoscopic images at a high frame rate and replays them at a slower speed, facilitating detailed observation of dynamic processes within the ET [7]. This advanced technique offers superior visualization of subtle structural changes compared to traditional endoscopy methods. Furthermore, DSVE has demonstrated improved diagnostic accuracy in identifying ETD. Studies have revealed significant differences in DSVE grades between ears affected with chronic otitis media and healthy ears, underscoring its potential as a diagnostic tool [7,8]. DSVE's ability to detect abnormalities that might evade detection with traditional techniques enhances its diagnostic utility. The DSVE findings add another layer of diagnostic precision where clinicians can glean valuable preoperative insights into ETD with other nasal and nasopharyngeal pathologies in one setting when combined with diagnostic nasal endoscopy [8]. This synergistic approach enhances the overall assessment and helps in assessing the effects of interventions on ET function; DSVE enables clinicians to monitor treatment progress and tailor patient management strategies accordingly [11]. This capability for longitudinal assessment contributes to more effective patient care and improved treatment outcomes.

DSVE: methodology

Overview of DSVE Procedure

The DSVE procedure employs slow-motion video analysis to visualize and evaluate dynamic structural alterations in the ET. Dynamic video endoscopy is valuable for visualizing and assessing the movements of the nasopharyngeal opening of the ET, aiding in diagnosing underlying pathologies in dysfunctional tubes. Grading ET function is crucial for predicting surgical outcomes for middle ear diseases. Employing slow-motion endoscopic video analysis, the DSVE procedure captures dynamic structural shifts in the ET, furnishing invaluable insights into the diagnosis and assessment of ETD [7]. Abnormal muscle action could stem from primary muscle weakness, inadequate insertion of the tensor veli palatini or dilator tubae, or a lack of coordinated effort between these muscles. Additionally, all cases of adhesive otitis media in our study exhibited ET dysfunction, reaffirming that ET dysfunction is a key factor in the pathogenesis of adhesive otitis media.

Equipment Required for DSVE

DSVE necessitates specific equipment tailored to visualize dynamic structural alterations within the ET. Typically, this includes an endoscope capable of capturing slow-motion video footage to facilitate precise observation of these changes. Depending on the preferences and specifications of the medical facility or research institution, the endoscope may be either flexible or rigid. Moreover, a high-quality video recording system is indispensable for meticulously documenting the endoscopic examination, enabling the recording of slow-motion footage for subsequent analysis [12]. Given the paramount importance of adequate lighting for clear visualization during endoscopic procedures, a compatible light source is invariably utilized with the endoscope. To facilitate real-time observation and analysis, a monitor is essential to display the live feed from the endoscope throughout the DSVE procedure. This enables medical professionals to scrutinize and interpret the dynamic behavior of the ET as it unfolds. In some instances, specialized software may complement the DSVE setup, facilitating the analysis of captured footage, measurement of dynamic structural changes, and precise interpretation of results [12,13].

Patient Preparation and Procedure Steps

Patient preparation for DSVE to evaluate ETD involves several essential steps to ensure thorough assessment and tailored treatment planning. During the DSVE procedure, the ET is meticulously visualized using slow-motion endoscopic video analysis to capture dynamic structural changes. Subsequently, DSVE grades are assigned to evaluate ET function in patients with COM and contralateral ears, which is considered normal. By comparing DSVE grades between ears with COM and normal ears, clinicians can discern significant differences, underscoring the potential of DSVE as a diagnostic tool for ETD [3]. Furthermore, DSVE is often integrated with other diagnostic tests as a comprehensive approach allows to assess ET function holistically. It helps healthcare providers obtain detailed information on the ET's structural and functional aspects, thereby enhancing ETD's diagnostic capabilities and guiding appropriate treatment strategies [14].

Video Recording and Analysis Techniques

Video recording and analysis techniques are integral components of DSVE for evaluating ETD. DSVE uses a rigid endoscope with specific characteristics, such as a 30-degree angle and 4 mm diameter, to visualize the nasopharyngeal area and the ET orifice. The patient is instructed to swallow saliva and breathe nasally throughout the procedure. At the same time, the endoscope captures the opening and closing of the ET, which is recorded in slow-motion video clips at 0.2-0.4 speed settings. These recordings of nasal endoscopy enable a meticulous analysis of dynamic structural changes within the ET which are scrutinized by experienced otolaryngologists, who assess the grade of ETD based on specific criteria [6,15]. This assessment includes evaluating factors such as the opening of the tubal lumen during swallowing and the presence of edema or congestion in the orifice mucosa [16,17].

Clinical applications of DSVE in ETD evaluation

Visualization of ET Function in Real-Time

The visualization of ET function in real-time has been explored across various imaging modalities. DSVE not only helps distinguish the type into mechanical and functional but also helps grade the ETD from normal to patulous under direct visualization. One notable study employed real-time MRI to observe the ET opening during the Valsalva maneuver in patients with confirmed dysfunction, shedding light on the dynamic function of the ET amidst specific maneuvers [1]. Furthermore, imaging techniques such as CT and MRI have proven effective in identifying features associated with obstructive or patulous ETD. However, comprehensive function assessments have been achieved with contrast-enhanced radiographs and scintigraphy, highlighting the potential for dynamic imaging to play a pivotal role in evaluating patients with ETD [18,19]. These findings underscore the significance of real-time imaging methods like MRI in capturing the dynamic function of the ET, providing valuable insights into its functionality during various physiological processes and in cases of dysfunction [1,18,20].

Identification of Specific ETD Pathologies

DSVE has emerged as a promising tool for identifying ETD pathologies. A study revealed that slow-motion endoscopic video analysis could be instrumental in categorizing types of pathologic changes in the ET [6,7]. In normal subjects, consistent muscle movement patterns were observed, contrasting with the absence of such patterns in abnormal subjects, indicating that DSVE offers valuable insights into ET anatomy and physiology [6,7]. DSVE was a valuable adjunct in providing information regarding the structural and functional status of the pharyngeal end of the ET [6,7]. This underscores the importance of effectively employing DSVE to identify specific ETD pathologies. Few studies have raised doubts over its efficacy as a standalone test, where authors felt that its utility is significantly enhanced when combined with other complementary diagnostic techniques [12,21]. However, some other recent studies have recommended the utility of DSVE as a single modality that has outstood other tests for ETD, as it has the added advantage of diagnosing the nasal, paranasal, and nasopharyngeal conditions associated with ETD at the same sitting [6,7,8]. The findings of ETD with signs and symptoms related to COM correlate better with DSVE than any other investigation alone [6,7,8,22]. This is also supported by the DSVE value being higher in COM ears than in contralateral ears with a higher grade of ETD [7,8].

Assessment of Mucosal Changes and Inflammation

The assessment of mucosal changes and inflammation in ETD is pivotal for comprehending the underlying pathophysiology of tubal dysfunction. DSVE has proven effective in evaluating these changes. DSVE not only helps in the assessment of the type of ETD, which is functional and mechanical but also helps in grading the ETD into grades 0,1,2,3, and patulous [6]. It helps identify and address the nasal, paranasal, and nasopharyngeal pathology affecting the ETD and correlates with it [6]. Research indicates a significant correlation between mucosal inflammation and ETD, underscoring the importance of assessing mucosal changes within the context of ETD evaluation [19]. Furthermore, DSVE plays a crucial role in grading ET movements based on the severity of tubal pathology depending on the mobility of the lumen, offering valuable insights into the structural and functional status of the ET [6,7]. The technique has been particularly beneficial in predicting outcomes after middle ear surgery, emphasizing its significance in clinical practice [7].

Correlation Between DSVE Findings and Patient Symptoms​​​​​​​

The correlation between DSVE findings and patient symptoms is important in evaluating conditions like ETD, nasal, paranasal, and nasopharyngeal pathology. Research indicates that DSVE effectively diagnoses ETD by analyzing the movements and structural changes of the ET, shedding light on the pathophysiology of tubal dysfunction [20]. Moreover, studies have revealed a substantial relationship between DSVE findings and middle ear disease, underscoring the necessity of aligning visual observations with patient symptoms [8,21,22]. Furthermore, DSVE plays a pivotal role in grading ET movements based on the severity of tubal pathology, establishing a direct connection between the visual assessment provided by DSVE and the symptoms experienced by patients with middle ear conditions [21]. By scrutinizing the dynamic structural changes in the ET, DSVE offers valuable insights into the functional status of the ET, potentially guiding treatment decisions based on the observed correlations between visual findings and patient-reported symptoms [8,23]. It helps to assess not only preoperative findings but also compare the improvement and effectiveness of the treatment in the postoperative period [6,24].

Comparative analysis: DSVE versus traditional methods

Accuracy and Reliability of DSVE​​​​​​​ Compared to Traditional Diagnostic Methods

DSVE stands out for its advantages in terms of accuracy and reliability when compared to traditional diagnostic methods. Accuracy, denoting the closeness of a measurement to the true value, and reliability, reflecting the consistency of measurements over time, are key metrics in assessing diagnostic techniques [25]. DSVE excels in both aspects by offering precise assessments by capturing dynamic structural changes in the ET, facilitating the diagnosis of ETD diagnosis, and predicting outcomes following middle ear surgery [6,26]. This accuracy is paramount in comprehending the pathophysiology of tubal dysfunction and guiding treatment decisions for patients with middle ear conditions [25,26,27]. Moreover, DSVE demonstrates reliability by providing consistent and repeatable measurements of ET movements, notwithstanding occasional inaccuracies due to factors such as systematic errors or instrument calibration issues [25]. The reliability of DSVE is underscored in studies correlating its findings with middle ear disease, illustrating its effectiveness in grading tubal movements based on the severity of tubal pathology [26]. Compared to traditional diagnostic methods, DSVE's combination of accuracy and reliability positions it as a superior tool for evaluating ETD. Its ability to offer precise and consistent measurements of ET function distinguishes it from conventional approaches, providing a more comprehensive and dynamic assessment that can significantly influence clinical decision-making and patient outcomes [25-28].

Cost-Effectiveness and Efficiency of DSVE

DSVE has proven cost-effective and efficient in evaluating ETD. By assessing dynamic structural changes in the ET and correlating with established diagnostic measures such as the Valsalva maneuver, DSVE offers a comprehensive evaluation of ETD [7]. DSVE enhances the preoperative assessment of ETD, demonstrating its cost-effectiveness in diagnosing and assessing the type and grading tubal movements based on the severity of tubal pathology [7]. This approach highlights the significance of evaluating the effectiveness and efficiency of diagnostic techniques like DSVE, ensuring optimal resource utilization and improved healthcare outcomes.

Patient Comfort and Safety Considerations

Patient comfort and safety considerations are paramount when employing DSVE to evaluate ETD. The research underscores DSVE's significance as an adjunct to impedance audiometry in assessing ET function, highlighting its role in diagnosing ETD and predicting outcomes following middle ear surgery [6,24]. Strong correlations between DSVE findings and middle ear disease emphasize its effectiveness in diagnosing ETD and grading tubal movements based on the severity of tubal pathology [6,24,29]. Regarding patient comfort, DSVE offers a non-invasive and well-tolerated method for evaluating ETD. The procedure entails transnasal endoscopic examination of the nasal, paranasal, and nasopharyngeal openings of the ET during rest, swallowing, and yawning, providing valuable insights into tubal movements and structural changes [27,29]. Regarding safety considerations, DSVE is deemed a safe diagnostic tool when conducted by trained professionals. The procedure enables visualization of dynamic structural changes in the ET without requiring invasive measures, reducing the associated risks compared to traditional diagnostic methods. Furthermore, DSVE furnishes valuable information about the structural and functional status of the ET, facilitating diagnosis and potentially influencing treatment decisions for patients with middle ear conditions [7,29]. This emphasis on patient comfort and safety underscores DSVE's role as a valuable and reliable diagnostic tool in evaluating ETD.

Current research and future directions

Recent Studies Utilizing DSVE for ETD Evaluation

Recent studies have utilized DSVE to evaluate ETD with promising outcomes. This study focused on grading ET movements based on DSVE observations to gauge the severity of tubal pathology [6]. In one retrospective study, patients diagnosed with chronic otitis media underwent surgery and were evaluated using DSVE alongside the Eustachian Tube Dysfunction Questionnaire (ETDQ-7). The findings revealed a significant correlation between DSVE results and intraoperative obstruction of the ET, underscoring the efficacy of DSVE in assessing preoperative ETD status [7]. Another research concluded that DSVE is a crucial tool in diagnosing ET dysfunction among patients with middle ear disease, highlighting the necessity for further investigations to explore DSVE's role in predicting outcomes following middle ear surgery [6,29]. These recent studies underscore the escalating significance of DSVE in ETD evaluation, offering valuable insights into ET's structural and functional changes. Moreover, they emphasize DSVE's potential to impact treatment decisions for patients with middle ear conditions [3,22].

Ongoing Research and Potential Advancements​​​​​​​ in DSVE Technology

Artificial intelligence (AI) holds tremendous potential in revolutionizing medical imaging technologies such as DSVE. By integrating AI algorithms, DSVE's analysis and interpretation of dynamic structural changes in the ET can be significantly enhanced. AI can improve the accuracy and efficiency of diagnosing ETD through DSVE imaging, potentially leading to more precise and timely diagnoses [30]. Innovations in data processing and visualization tools offer further opportunities to enhance DSVE analysis. By leveraging advanced data processing techniques, researchers and clinicians can conduct more detailed assessments of tubal movements and structural changes observed in DSVE images. Real-time data processing capabilities improve diagnostic accuracy and expedite the evaluation process, thereby enhancing the efficiency of DSVE evaluations [30]. The development of smart devices, including advanced cameras and sensors, is poised to elevate the quality and precision of DSVE imaging. Integrating these smart devices with DSVE technology equips researchers and clinicians with sophisticated tools for capturing and analyzing dynamic movements in the ET. This integration holds promise for enhancing the overall capabilities of DSVE, leading to improved diagnostic outcomes and better-informed treatment decisions [31].

Integration of DSVE Into Routine Clinical Practice

The integration of DSVE into routine clinical practice for evaluating ETD represents a promising approach with significant potential to enhance the diagnosis and management of this condition [6,32,33]. This integration of clinical tools streamlines the diagnostic process and facilitates informed treatment decisions for patients with ETD [27,32,33]. Furthermore, DSVE has demonstrated utility in predicting outcomes following middle ear surgery, underscoring its importance in clinical practice where septoplasty can be one of the treatment plans for middle ear pathology where the causative factor is ETD due to deviated nasal septum. This may help in better prognosis and better outcomes of graft approval on a long-term basis [22,27,32]. Through incorporating DSVE into routine clinical workflows, healthcare providers gain enhanced capabilities to assess tubal function, grade the severity of tubal pathology, and make well-informed decisions regarding surgical interventions [32]. To successfully integrate DSVE into routine clinical practice, a coordinated effort involving various stakeholders within the healthcare system is imperative [33]. This may entail establishing standardized protocols for DSVE acquisition and interpretation, integrating DSVE findings into electronic health records, and providing comprehensive training and education to healthcare professionals on the utilization and interpretation of DSVE [33]. Such concerted efforts ensure the seamless integration of DSVE into clinical practice, thereby optimizing patient care and outcomes in evaluating and managing ETD.

## Conclusions

In conclusion, DSVE emerges as a transformative tool in evaluating ETD. Literature findings underscore its efficacy in enhancing diagnostic accuracy by enabling direct observation of ET function and facilitating the identification of specific pathologies contributing to ETD symptoms. Further research is needed to assess the utility of DSVE as a standalone and gold standard test for diagnosing eustachian dysfunction, though most of the studies seem to recommend DSVE as a highly useful tool for the assessment of ETD.
